# Gsformer: a dual-architecture deep learning framework with CNN-self-attention and sparse-attention for genomic selection

**DOI:** 10.1186/s12711-026-01055-8

**Published:** 2026-06-03

**Authors:** Tingting Yang, Weicong Wu, Yahui Xue, Lei Zhou, Huimin Kang, Jianfeng Liu

**Affiliations:** 1 Guangdong Provincial Key Laboratory of Animal Molecular Design and Precise Breeding, School of Animal Science and Technology, Foshan University, Foshan, 528000 China; 2 State Key Laboratory of Animal Biotech Breeding, Frontiers Science Center for Molecular Design Breeding (MOE), College of Animal Science and Technology, China Agricultural University, Beijing, 100193 China

## Abstract

**Background:**

Genomic selection (GS) has revolutionized modern breeding by utilizing genome-wide single nucleotide polymorphisms (SNPs). While traditional models such as GBLUP and Bayesian approaches remain prevalent, several deep learning approaches have recently been introduced for plant GS, demonstrating superior predictive performance. Here, we introduce Gsformer, a novel deep learning framework designed to predict phenotypes by modeling complex genetic architectures. It features two distinct architectures: CSA, which combines convolutional neural networks (CNNs) with self-attention to capture local and long-range genomic dependencies, and NSA, which employs a native sparse attention mechanism to enhance computational efficiency by focusing on the most informative features. We evaluated Gsformer on six datasets spanning animal and plant species—pig, cattle, chicken, mouse, wheat, and maize—and compared its phenotypic prediction performance against five established GS methods: DNNGP, MLP, LightGBM, SVR, and GBLUP.

**Results:**

Gsformer generally ranked among the top two models across six diverse animal and plant genomic prediction datasets. Specifically, Gsformer-CSA yielded notable improvements in predicting cattle fat percentage, while Gsformer-NSA was more accurate in predicting chicken first egg weight, pig age at 100 kg body weight, and mouse anxiety. With the topN hyperparameter set to 20%, Gsformer-NSA matched or marginally exceeded Gsformer-CSA for most traits—though it showed lower accuracy for a subset of traits. Adjusting the topN value further enhanced Gsformer-NSA’s performance, allowing it to match that of Gsformer-CSA. Ablation studies confirmed the complementary roles of CNN and self-attention modules in the CSA architecture. To enhance interpretability, we applied SHAP (SHapley Additive exPlanations) to identify influential SNPs and annotate candidate genes associated with growth and body size traits in pigs. Functional enrichment analysis revealed biologically relevant pathways involved in nervous system development, glycolytic process regulation, and digestive tract morphogenesis.

**Conclusions:**

In summary, Gsformer establishes a flexible and powerful framework for genomic prediction, demonstrating broad applicability across both animal and plant breeding. Owing to its lower computational cost, Gsformer-NSA is recommended over Gsformer-CSA in scenarios where the minor sacrifice in prediction accuracy is acceptable.

**Supplementary Information:**

The online version contains supplementary material available at 10.1186/s12711-026-01055-8.

## Background

Genomic selection (GS), first introduced by Meuwissen et al. [1], leverages genome-wide molecular marker information to estimate breeding values. By accurately evaluating individual breeding potential, GS improves selection accuracy, shortens generation intervals, and significantly accelerates genetic progress [2]. Over the past decade, substantial reductions in genotyping costs have facilitated widespread adoption of GS, yielding considerable increases in genetic improvement in both animal and plant breeding programs [3, 4].

Statistical models for GS can be broadly categorized into three groups: best linear unbiased prediction (BLUP)-based methods, Bayesian approaches, and machine learning techniques. BLUP-based methods treat breeding values as random effects and employ a relationship matrix, derived from the genetic information of reference and candidate populations, which serves as the variance–covariance structure for estimating variance components and computing genomic estimated breeding values (GEBVs) [5]. Prominent examples include genomic BLUP (GBLUP) [6], and single-step GBLUP (ssGBLUP) [7, 8]. Bayesian methods explicitly estimate marker effects using phenotypic and genotypic data from the reference population, with different models making varying assumptions about the distribution of these effects. Examples include BayesA [1], BayesB [9], BayesC [10], and Bayesian LASSO [11]. While these traditional GS prediction models rely on strong parameter assumptions and linear regression frameworks, they often fail to capture the nonlinear relationships inherent in high-dimensional genomic data.

In contrast, machine learning (ML) methods are model-agnostic: they learn genotype–phenotype mappings directly from data, without assuming specific distributions of marker effects or predefining genetic architecture. As a result, they naturally encode non-additive effects—including dominance and epistasis—as well as genotype × environment interactions, all within a single predictive framework [12–14]. Nevertheless, mainstream GS models—including GBLUP, BayesA, and Bayesian LASSO—rely exclusively on additive SNP effects and thus ignore non-additive variation. Empirical evidence, however, shows that non-additive effects contribute substantially to phenotypic variation, underpin heterosis in hybrid programs, and significantly improve prediction accuracy of certain traits [15–17]. Consequently, ML techniques such as support vector regression (SVR) [18], kernel ridge regression (KRR) [19], LightGBM [20], and deep learning (DL) [21] are increasingly adopted in GS and have demonstrated superior prediction performance across diverse traits and species.

Among ML techniques, DL has garnered significant and rapidly growing attention in GS applications. For plant breeding, models such as DeepGS [22] and DNNGP [23], built on convolutional neural network (CNN) architectures, and have demonstrated superior predictive performance across multiple datasets compared to conventional GS methods. Moreover, GPformer [24], which incorporates a variant of the self-attention mechanism along with a knowledge-guided module, has further improved prediction accuracy. Recently, Cropformer [25], combining predictive strengths of CNNs with a self-attention mechanism, achieved significant advances for crop species.

Despite these advances, most existing DL frameworks for GS have been primarily designed for plant applications. Additionally, the computational complexity associated with the standard self-attention mechanism poses a significant challenge when handling high-dimensional genomic data. Native sparse attention [26] offers a promising solution to this limitation, reducing computational demands while preserving, or even enhancing, model performance.

To address these challenges, we developed Gsformer, a deep learning model for genomic selection that provides two distinct feature extraction strategies: CSA (CNN with Self-Attention) and NSA (Native Sparse Attention). Specifically, CSA integrates CNNs with a self-attention mechanism, whereas NSA leverages native sparse attention to efficiently capture relevant genomic features. By capturing non-linear and interaction effects, both strategies enable the model to estimate total genetic merit for phenotypic prediction. To enhance model interpretability, we applied SHapley Additive exPlanations (SHAP) to quantify the contribution of individual input features to the final predictions. We comprehensively assessed Gsformer's predictive performance through extensive comparative experiments against five widely used genomic selection methods—DNNGP, MLP, LightGBM, SVR, and GBLUP—using both animal and plant genomic datasets.

## Methods

### Overview of Gsformer

**Fig. 1:**
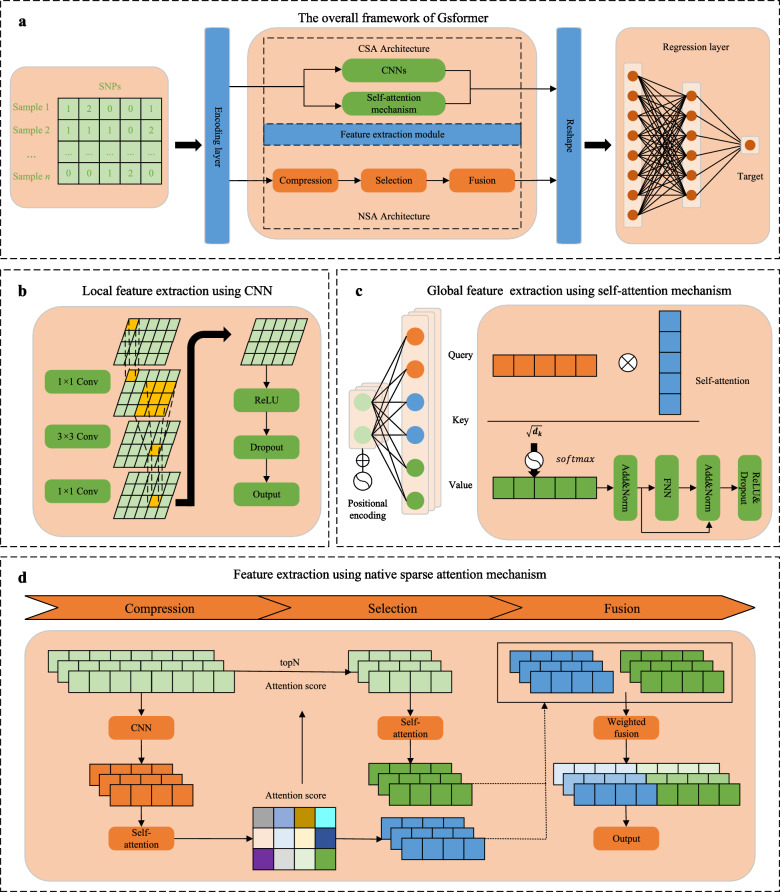
Architecture overview of Gsformer. **a** The overall framework of Gsformer, which consists of an encoding layer, a feature extraction module, and a regression layer. The input is genomic data, represented either in 01-encoded or dimensionality-reduced form. **b** The local feature extraction component in Gsformer-CSA, where each sample is processed through three consecutive convolutional layers (CNNs) to capture local dependencies. **c** The global feature extraction component in Gsformer-CSA, which employs a multi-head self-attention mechanism to model long-range dependencies within the genomic data. **d** Feature extraction in the Gsformer-NSA, which implements a sparse workflow through compression, selection, and fusion stages

The architecture of Gsformer, illustrated in Fig. 1a, comprises three main components: an encoding layer, a feature extraction module, and a regression layer. In the encoding layer, single nucleotide polymorphism (SNP) markers are transformed into high-dimensional matrix representations. These representations are then fed into the feature extraction module, which offers two alternative strategies—CSA (CNN with Self-Attention) and NSA (Native Sparse Attention)—for extracting genomic features. The resulting features are reshaped and passed to the regression layer, which predicts phenotypic values using two linear layers separated by an activation function and dropout regularization. Layer normalization is applied to the regression layer input to enhance training stability. Model performance in regression tasks is evaluated using mean squared error (MSE) [27].

#### Feature extraction module based on the CSA architecture

The CSA architecture integrates CNNs and a self-attention mechanism in a parallel manner, enabling the model to jointly capture both local and global patterns from the input data. The CNN subnetwork consists of three stacked convolutional layers with kernel sizes of 1 × 1, 3 × 3, and 1 × 1, respectively (Fig. 1b). This is followed by a ReLU activation function and dropout regularization to reduce overfitting. Input and output channel dimensions are consistently maintained at 1, with a fixed stride of 1 throughout. To preserve spatial resolution, zero-padding is applied in the 3 × 3 convolutional layer, ensuring that no critical structural information is lost during feature extraction. The use of multi-scale kernels enables complementary feature learning: the 1 × 1 kernels refine local feature representations, while the 3 × 3 kernels capture broader contextual patterns.

For global feature modeling, the architecture based on self-attention mechanism composed of the following components: positional encoding (implemented using sine and cosine functions [28]) to preserve sequential order; multi-head self-attention for long-range dependency modeling; feedforward neural networks (FNNs) enabling non-linear transformation; normalization layers (Norm) with residual connections to enhance training stability and prevent gradient vanishing; as well as dropout regularization to reduce overfitting (Fig. 1c).

Within the multi-head self-attention mechanism, the input feature matrix **X** is linearly projected into Query **Q**, Key **K**, and Value **V** matrices through three learned weight matrices ${\mathbf{W}}_{{\mathrm{Q}}}$, ${\mathbf{W}}_{{\mathrm{K}}}$, and ${\mathbf{W}}_{{\mathrm{V}}}$, respectively:

1 $$ {\mathbf{Q}} = {\mathbf{XW}}_{{\mathrm{Q}}} , $$

2 $$ {\mathbf{K}} = {\mathbf{XW}}_{{\mathrm{K}}} , $$

3 $$ {\mathbf{V}} = {\mathbf{XW}}_{{\mathrm{V}}} . $$

Each attention head independently computes scaled dot-product attention:

4 $$ {\mathbf{head}}_{i} = Attention\left( {{\mathbf{Q}},{\mathbf{K}},{\mathbf{V}}} \right) = softmax\left( {\frac{{{\mathbf{QK}}^{T} }}{{\sqrt {d_{k} } }}} \right){\mathbf{V}}, $$

where $d_{k}$ is the dimension of the **K** matrix, used to scale the dot product and prevent gradient vanishing in high-dimensional spaces.

The outputs from all attention heads are then concatenated and transformed through a final linear projection layer to generate the output representation:

5 $$MultiHead\left({\mathbf{Q}},{\mathbf{K}},{\mathbf{V}}\right) = Concat\left( {{\mathbf{head}}_{1} , \ldots ,{\mathbf{head}}_{n} } \right){\mathbf{W}}_{{\mathrm{O}}} , $$

where *n* is the number of attention heads, and ${\mathbf{W}}_{{\mathrm{O}}}$ is the learnable weight matrix.

#### Feature extraction module based on the NSA architecture

The NSA architecture employs native sparse attention mechanism to efficiently extract features while reducing computational complexity (Fig. 1d). Its feature extraction process follows a three-stage workflow: compression, selection, and fusion. During compression, the input sequence is condensed into a compact representation to reduce computational load. The selection stage then identifies the most informative regions of the original input using this compressed representation. Finally, in the fusion stage, the outputs from both stages are fused using weighted gated scoring and fed into the regression layer for downstream predictions.

More specifically, the compression stage focuses on coarse-grained processing of the input data. The compressed attention mechanism begins by dividing the input into consecutive blocks, where Keys (**K**) and Values (**V**) within each block are aggregated into block-level representations ${\tilde{\mathbf{K}}}_{t}^{cmp}$ and ${\tilde{\mathbf{V}}}_{t}^{cmp}$ using CNN for compression, respectively:

6 $${\tilde{\mathbf{K}}}_{t}^{cmp} = \left\{ {\varphi \left( {{\mathbf{k}}_{id + 1:id + l} } \right) \mid 1 \le i \le \left\lfloor \frac{t - l}{d}\right\rfloor} \right\}, $$

7 $${\tilde{\mathbf{V}}}_{t}^{cmp} = \left\{ {\varphi \left( {{\mathbf{v}}_{id + 1:id + l} } \right) \mid 1 \le i \le \left\lfloor\frac{t - l}{d} \right\rfloor} \right\}, $$

where *t* is the length of the genomic data within each block; *l* represents the number of tokens contained in each compressed block; *d * denotes the sliding step, which refers to the overlap step between adjacent compressed blocks; and *φ* is a learnable MLP with intra-block position encoding to map keys in a block to a single compressed key [26].

Subsequently, attention scores are computed based on the compressed keys and queries, accurately reflecting the relevance of each compressed block to the current query:

8 $$ {\mathbf{p}}_{t}^{cmp} = softmax\left( {{\mathbf{Q}}_{t}^{T} {\tilde{\mathbf{K}}}_{t}^{cmp} } \right). $$

The selected attention mechanism enhances feature representation by focusing on fine-grained details. Specifically, based on the attention scores obtained through compressed attention, importance weights are assigned to each block, and the topN most relevant blocks are selected. Subsequently, rotary position encoding [29] is applied to the Key and Query matrices of the original token-level features within the selected blocks. The standard attention computation is then performed.

Beyond differences in their core feature extraction approaches, the CSA and NSA architectures also utilize distinct normalization techniques and activation functions. Specifically, the CSA model employs Layer Normalization along with ReLU activation, whereas the NSA model utilizes RMSNorm and GELU activation. To enhance the generalization capability of Gsformer and alleviate overfitting, we integrated a set of regularization techniques during the training process, including cross-validation, L2 regularization, and early stopping. Cross-validation is implemented by partitioning the dataset into multiple disjoint subsets (folds), followed by iterative training and validation across these folds to ensure robust and consistent performance on unseen data. L2 regularization constrains the magnitude of model weights to reduce model complexity, while early stopping monitors validation performance and terminates training when no further improvement is observed, thereby preserving computational resources.

### Datasets

We analyzed datasets spanning animal and plant species. With the exception of the pig dataset, all other datasets were obtained from public repositories or published studies: chicken [30], cattle [31], mouse [32], wheat [33], and maize [34]. Genotypes were encoded as follows: heterozygous genotypes were assigned a value of 1, while homozygous genotypes were encoded as 0 or 2. The wheat and maize datasets were used directly as reported in the DNNGP study [23]. For the remaining datasets, quality control was performed using PLINK v1.90 [35] with the following criteria: individual call rate ≥ 95%, genotype call rate > 95%, minor allele frequency (MAF) > 0.01, Hardy–Weinberg equilibrium (HWE) test *P*-value ≥ 1.0 × 10⁻^6^, and inclusion of autosomal SNPs only. The MAF threshold was set to > 0.01 for most datasets; however, for the mouse dataset, which has a relatively small sample size (*n* = 327), we applied a more stringent threshold of MAF > 0.05. This adjustment was made because lower MAF thresholds in small samples can lead to unreliable allele frequency estimates, reduced statistical power, and amplification of genotyping errors. Elevating the threshold enhances the robustness of the analysis, aligning with quality control practices for small-scale genomic studies [36, 37].

As described in the DNNGP study [23], principal component analysis (PCA) [38] was performed prior to genomic prediction to reduce the dimensionality of the SNP data, retaining 95% of the total genetic variance for subsequent DNNGP-based GS. For the maize dataset, all methods used the resulting principal components as input features. In contrast, for the wheat dataset and all animal datasets, PCA was applied only to the DNNGP method; all other methods were trained and evaluated directly using SNP genotypes. PCA is widely applied for dimensionality reduction in ML-based genomic prediction. Notably, several machine learning methods—including DNNGP—achieved comparable prediction accuracy with and without PCA preprocessing [23]. To assess whether the asymmetry in input preprocessing—PCA projection for DNNGP versus raw SNP genotypes for all other methods—confounds cross-method performance comparisons, we re-ran DNNGP without PCA on two representative traits: anxiety (Anx) (mouse dataset) and GY3 (wheat dataset).

The pig dataset consisted of 2,162 Yorkshire pigs from a commercial breeding company in China. Traits included age adjusted to 100 kg body weight (AGE), backfat thickness adjusted to 100 kg (BF), and three conformation traits measured at approximately 150 days of age: body length (Length), body height (Height), and chest circumference (CC). Corrected phenotypes were computed using best linear unbiased prediction (BLUP), defined as the sum of estimated breeding values (EBVs) and residuals. For the growth traits, the precorrection models included herd-year-season and sex as fixed effects, and litter effect as a random effect; for the conformation traits, herd-year-season, sex, and age in months were used as fixed effects. The corrected phenotypes were used as response variables in GS. Genotyping was performed using the KPSISUS50-V1 chip containing 43,832 SNPs. After quality control, 35,495 SNPs and 1,191 genotypic principal components (PCs) were retained for GS analysis.

The chicken dataset originated from a Rhode Island Red population maintained by Beijing Huadu Yukou Poultry Breeding Co., Ltd., comprising 1,063 individuals and 294,705 SNPs. Analyzed traits included first egg weight (FEW), egg weight at 28 weeks (EW28), and at 36 weeks (EW36). All SNPs passed quality filtering. To balance marker density with computational efficiency while preserving genome-wide coverage, we implemented a uniform SNP thinning strategy [39, 40], a method widely adopted in genomic selection studies to reduce redundant linkage disequilibrium (LD) and alleviate computational burden without significantly compromising prediction accuracy [41, 42]. Specifically, we performed SNP thinning using non-overlapping windows of nine consecutive SNPs, selecting only the fifth SNP from each window. This resulted in 32,745 SNPs, from which 681 PCs were extracted for GS.

The cattle dataset was obtained from Vereinigte Informationssysteme Tierhaltung w.V. and included 5,024 German Holstein bulls genotyped using the Illumina Bovine SNP50 BeadChip [43] (42,551 SNPs). EBVs for milk yield (MY), fat percentage (FP), and somatic cell score (SCS) were used as response variables in GS. For computational efficiency, only the first 2500 samples were used, yielding 1,716 PCs after PCA.

The mouse dataset contained genotypic and phenotypic records for 327 outbred mice. Traits analyzed included Anx, emotionality (Emo), and blood glucose level (BGL). Following quality control, 8,211 SNPs were retained, and 224 PCs were derived for subsequent analysis.

The wheat dataset was sourced from the CIMMYT public repository and included grain yield (GY) data for 599 varieties across four environments (GY1–GY4). Genotype information consisted of 1279 markers and 251 PCs were extracted from them via PCA. As described in the DNNGP study [23], environmental noise was removed from the phenotypic data, and EBVs were computed using a mixed linear model for method evaluation.

The maize dataset comprised 1,404 progeny that descended from 24 Chinese elite inbred maize. Based on data provided in the DNNGP study [23], this dataset included 1,056 PCs derived from genomic data and four agronomic traits: days to anthesis (DTA), plant height (PH), kernel number per ear (KNPE), and kernel weight per ear (KWPE). All GS methods utilized the 1,056 PCs for genomic prediction.

### Model evaluation

To evaluate the predictive performance of the Gsformer model with two feature extraction architectures—CSA and NSA (hereafter referred to as Gsformer-CSA and Gsformer-NSA, respectively)—we conducted comparative experiments against a range of widely used machine learning and genomic prediction models, including DNNGP, MLP, LightGBM, SVR, and GBLUP, across six datasets. We employed a five-fold outer / three-fold inner nested cross-validation scheme to partition the training data [44]. The inner loop was used for hyperparameter optimization via grid search, while the outer loop provided an unbiased estimate of model generalization performance. The final prediction accuracy for each model was calculated as the average Pearson correlation coefficient between predicted values and: (1) corrected phenotypes for the pig dataset, (2) EBVs for the cattle, wheat, and maize datasets, or (3) observed phenotypes for the remaining datasets.

### Features contribution analysis

To interpret the contribution of individual input features to model predictions, we employed SHAP, a model-agnostic interpretability framework widely adopted for feature importance analysis [45]. In this framework, the absolute SHAP value quantifies the magnitude of a feature’s influence on a given prediction, with higher values indicating stronger influence. As a representative case to illustrate the integration of SHAP with the Gsformer model for identifying potential candidate genes, we applied SHAP analysis to the pig dataset to evaluate the contribution of each input feature to the response variable.

Specifically, we performed SHAP analysis using the Gsformer-NSA model to identify the top 10 SNPs with the highest SHAP values for each trait in the pig dataset. Due to computational memory limitations, the SHAP analysis was performed using 50 background samples and 500 explanation samples, which were randomly selected to ensure representativeness. SHAP values were computed based on these samples. Based on the pig reference genome *Sus scrofa* 10.2, we defined gene regions located within 100 kb upstream and downstream of each selected SNP. Candidate genes were annotated using the BEDTools software suite [46]. Subsequently, Gene Ontology (GO) and Kyoto Encyclopedia of Genes and Genomes (KEGG) pathway enrichment analyses were performed on the annotated genes via the KOBAS online platform (http://bioinfo.org/kobas/genelist/). The statistical significance of enrichment was evaluated using Fisher’s exact test. To account for multiple hypothesis testing, *p*-values were adjusted using the Benjamini–Hochberg procedure, and results with a corrected *p*-value < 0.05 were considered significantly enriched.

## Results

### Prediction accuracy of Gsformer and comparison with other methods

We evaluated the GS accuracy of Gsformer-CSA and Gsformer-NSA in comparison with five widely used statistical and ML methods—DNNGP, MLP, LightGBM, SVR, and GBLUP—using six animal and plant datasets. The hyperparameter values used in the grid search for the ML methods are listed in Additional file 1 Table S1.

**Fig. 2:**
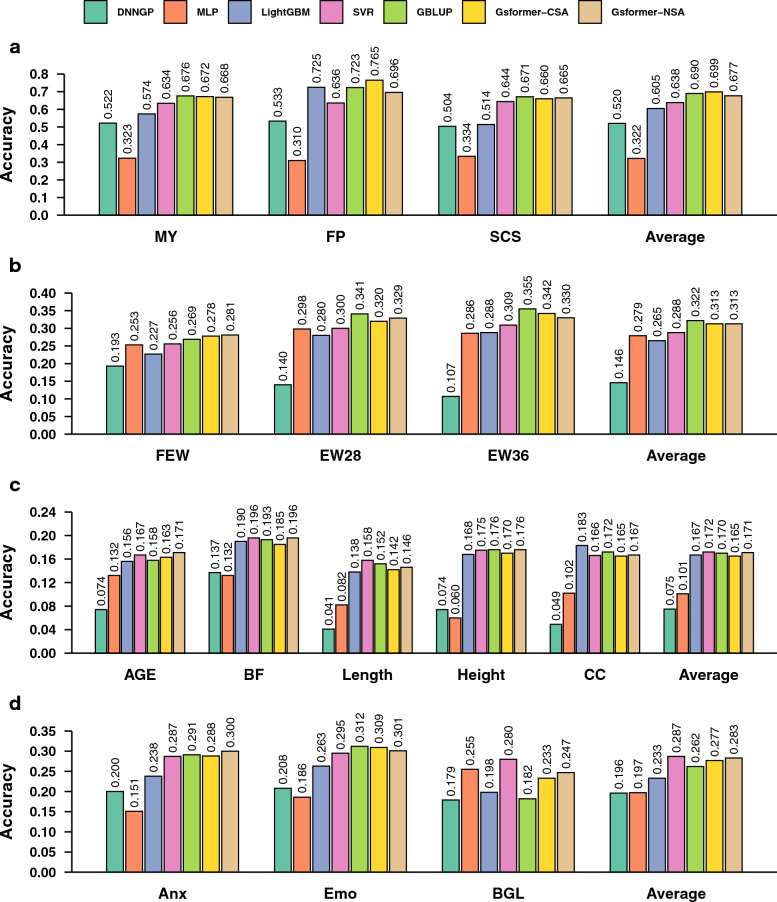
Prediction accuracy comparison of seven methods on the animal datasets. The x-axis lists different traits, with each trait evaluated by seven methods (from left to right: DNNGP, MLP, LightGBM, SVR, GBLUP, Gsformer-CSA, and Gsformer-NSA). **a** Cattle dataset: milk yield (MY), fat percentage (FP), and somatic cell score (SCS). **b** Chicken dataset: first egg weight (FEW), egg weight at 28 weeks (EW28), and egg weight at 36 weeks (EW36). **c** Pig dataset: age at 100 kg body weight (AGE), backfat thickness at 100 kg (BF), and three conformation traits at 150 days of age—body length (Length), body height (Height), and chest circumference (CC). **d** Mouse dataset: anxiety (Anx), emotionality (Emo), and blood glucose level (BGL)

The predictive performance of the models on animal datasets is shown in Fig. 2. On the cattle dataset, prediction accuracies across the three milk-related traits (MY, FP, and SCS) exhibited consistent trends among models (Fig. 2a). Among the seven compared methods, Gsformer-CSA achieved the highest prediction accuracy for FP (0.765), outperforming the second-best method (0.725) by a substantial margin of 0.04. For both MY and SCS, GBLUP achieved the highest prediction accuracy—0.676 for MY and 0.671 for SCS—marginally outperforming the second-best models: Gsformer-CSA (0.672 for MY) and Gsformer-NSA (0.665 for SCS), respectively. Based on the mean accuracy across all three traits, Gsformer-CSA emerged as the most accurate model, whereas MLP performed the worst. Specifically, Gsformer-CSA outperformed GBLUP, Gsformer-NSA, SVR, LightGBM, DNNGP, and MLP by 1.30%, 3.25%, 9.56%, 15.54%, 34.42%, and 117.08%, respectively.

On the chicken dataset, GBLUP attained the highest mean prediction accuracy across the three egg production traits—FEW, EW28, and EW36—(0.322), while Gsformer-CSA and Gsformer-NSA achieved virtually identical and comparably high accuracies of 0.313 (Fig. 2b). On average, GBLUP outperformed Gsformer-CSA, Gsformer-NSA, SVR, LightGBM, DNNGP, and MLP by 2.88%, 2.88%, 11.81%, 21.51%, 15.41%, and 120.55%, respectively. Specifically, Gsformer-NSA performed best on FEW, while GBLUP showed superior accuracy for EW28 and EW36.

On the larger pig dataset, predictive performance was evaluated across five growth- and body size-related traits. Based on mean prediction accuracy across all traits (Fig. 2c), SVR achieved the highest accuracy (0.172), followed closely by Gsformer-NSA (0.171) and GBLUP (0.170). Relative to Gsformer-NSA, GBLUP, LightGBM, Gsformer-CSA, DNNGP, and MLP, SVR exhibited average accuracy improvements of 0.58%, 1.18%, 3.00%, 4.24%, 70.30%, and 129.33%, respectively. Trait-specific prediction accuracy varied: Gsformer-NSA achieved the highest accuracy for AGE, BF, and Height; SVR for Length (tied with Gsformer-NSA for BF); LightGBM for CC; and GBLUP for Height (tied with Gsformer-NSA).

On the mouse dataset, Gsformer-NSA, GBLUP, and SVR achieved the highest prediction accuracy for Anx, Emo, and BGL, respectively (Fig. 2d). SVR demonstrated statistically superior overall predictive performance: it outperformed Gsformer-NSA, Gsformer-CSA, GBLUP, LightGBM, DNNGP, and MLP by 1.41%, 3.61%, 9.54%, 23.18%, 45.69%, and 46.43% in mean accuracy, respectively.

**Fig. 3:**
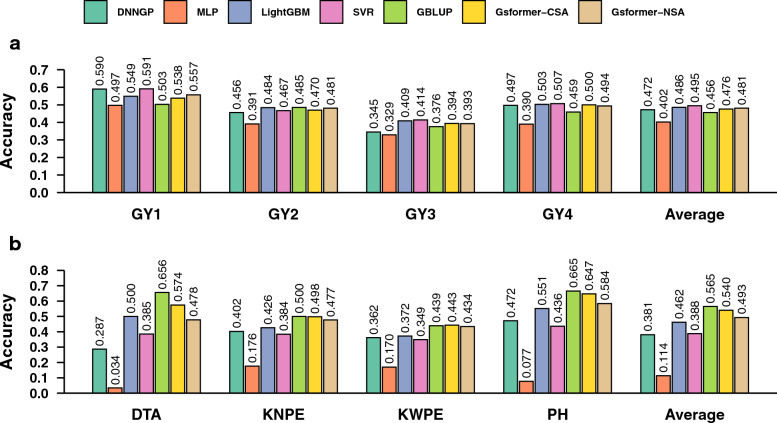
Prediction accuracy comparison of seven methods on the plant datasets. The *x*-axis lists different traits, with each trait evaluated by seven methods (from left to right: DNNGP, MLP, LightGBM, SVR, GBLUP, Gsformer-CSA, and Gsformer-NSA). **a** Wheat dataset: grain yield (GY) across four distinct environments (GY1, GY2, GY3, GY4). **b** Maize dataset: days to anthesis (DTA), plant height (PH), kernel number per ear (KNPE), and kernel weight per ear (KWPE)

In addition to the evaluation on animal datasets, we further assessed the performance of Gsformer using two plant datasets (wheat and maize) (Fig. 3). On the wheat dataset, mean prediction accuracy across the four traits (GY1, GY2, GY3, and GY4) revealed that SVR achieved the highest overall performance, followed closely by LightGBM and Gsformer-NSA (Fig. 3a). Relative to LightGBM, Gsformer-NSA, Gsformer-CSA, DNNGP, GBLUP, and MLP, SVR and showed mean accuracy improvements of 1.85%, 2.91%, 3.99%, 4.87%, 8.55%, and 23.13%, respectively. For mean prediction accuracy per trait, SVR achieved the highest accuracy on GY1, GY3, and GY4, whereas GBLUP performed best on GY2.

On the maize dataset, prediction accuracy varied substantially across methods for DTA and PH. GBLUP achieved the highest accuracy on DTA, KNPE, and PH; Gsformer-CSA ranked second for all three traits. In contrast, Gsformer-CSA attained the top accuracy on KWPE. On average, GBLUP achieved the highest accuracy across all four traits, surpassing Gsformer-CSA, Gsformer-NSA, LightGBM, SVR, DNNGP, and MLP by 4.63%, 14.60%, 22.29%, 45.62%, 48.29%, and 395.61%, respectively (Fig. 3b).

### Adjusting topN enables Gsformer-NSA to match Gsformer-CSA performance

In our previous analyses, the topN parameter—the proportion of highest-scoring SNPs retained as input features—was fixed at 20%, based on empirical observation of signal-to-noise balance in pilot analyses. Although topN governs model sparsity and may indirectly affect predictive capacity, it was treated as a fixed architectural design choice, not a hyperparameter subject to optimization, to avoid conflating feature selection strategy with model tuning.

To rigorously assess whether alternative sparsity levels could narrow the performance gap between Gsformer-NSA and Gsformer-CSA—particularly for traits where the former exhibited lower accuracy—we performed a controlled sensitivity analysis on FP (cattle) and DTA (maize). For each, we evaluated topN values of 20%, 40%, 60%, 80%, and 100%. All analyses employed nested cross-validation with a five-fold outer loop (for unbiased estimation of generalization performance) and a three-fold inner loop (for hyperparameter optimization); critically, topN was pre-specified prior to any cross-validation and remained invariant throughout all outer and inner folds.

**Fig. 4:**
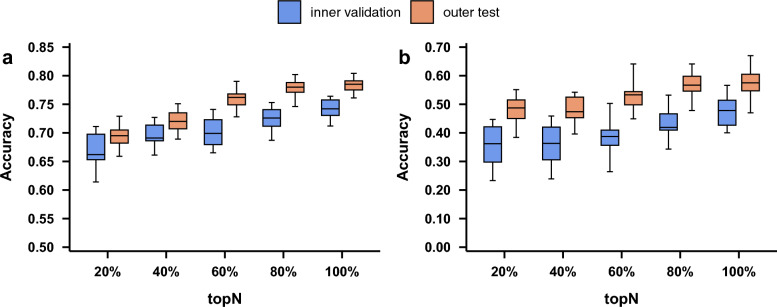
Impact of topN on Gsformer-NSA prediction accuracy under five-fold outer / three-fold inner nested cross-validation. **a** Fat percentage. **b** Days to anthesis

As shown in Fig. 4, the prediction accuracy of Gsformer-NSA consistently improved on both the inner validation and outer test sets as the topN value increased. For the FP trait, increasing topN from 20 to 60% raised the outer-test accuracy from 0.697 to 0.760. A further increase to 80% achieved an accuracy of 0.778, matching the performance of Gsformer-CSA on this trait. Similarly, for the DTA trait, increasing topN from 20 to 100% improved the outer-test accuracy from 0.478 to 0.578, again matching Gsformer-CSA. These results demonstrate that through tuning the sparsity level (topN), Gsformer-NSA can achieve prediction accuracy comparable to that of Gsformer-CSA.

### Ablation analysis of Gsformer-CSA

To evaluate the individual contributions of Gsformer-CSA's components within the CSA architecture, we conducted an ablation study quantifying the impact of its self-attention mechanism (SAM) and CNN component on predictive performance. We compared three configurations: the full Gsformer-CSA model, a SAM-only variant, and a CNN-only variant.

As shown in Table 1, the full model significantly outperformed both reduced variants in prediction accuracy. Notably, the SAM-only model achieved higher accuracy than the CNN-only model, demonstrating the self-attention mechanism's superior feature extraction capability. Conversely, the CNN module provided complementary information that enhanced overall predictive performance. The integration of both modules in Gsformer-CSA thus leverages their synergistic strengths, enabling robust feature extraction and achieving state-of-the-art performance in genomic prediction tasks.

**Table 1:** Prediction accuracy of Gsformer-CSA and its variants in the ablation analysis using animal datasets

Dataset	Trait^1^	The full Gsformer-CSA model	SAM-only variant^2^	CNN-only variant^3^
Cattle	MY	0.672	0.654	0.661
	FP	0.765	0.764	0.743
	SCS	0.660	0.649	0.656
Chicken	FEW	0.278	0.269	0.256
	EW28	0.320	0.313	0.233
	EW36	0.342	0.276	0.296
Pig	AGE	0.163	0.154	0.159
	BF	0.185	0.176	0.184
	Length	0.142	0.140	0.141
	Height	0.170	0.170	0.132
	CC	0.165	0.153	0.164
Mouse	Anx	0.288	0.284	0.243
	Emo	0.309	0.275	0.246
	BGL	0.233	0.205	0.201

^1^MY: milk yield; FP: fat percentage; SCS: somatic cell score; FEW: first egg weight; EW28: egg weight at 28 weeks; EW36: egg weight at 36 weeks; AGE: age adjusted to 100 kg body weight; BF: backfat thickness adjusted to 100 kg; Length: body length at 150 days old; Height: body height at 150 days old; CC: chest circumference at 150 days old; Anx: anxiety; Emo: emotionality; BGL: blood glucose level

^2^SAM-only variant: self-attention mechanism (SAM)-only variant of Gsformer-CSA model

^3^CNN-only variant: convolutional neural network (CNN)-only variant of Gsformer-CSA model

### Identification of candidate genes for growth and body size traits in the pig dataset

Using SHAP analysis, we identified the top 10 SNPs with the highest contribution to each trait based on their SHAP values in the pig dataset. Within a 100 kb region flanking these SNPs, we annotated 15, 11, 12, 15, and 6 candidate genes associated with AGE, BF, Length, Height, and CC, respectively (Additional file 1 Table S2). Several of these genes have been previously reported to be associated with similar traits in the Animal QTL Database (accessed 20 Jul 2025) [47]. These include *PRIM2* (associated with average daily gain, carcass length, femur length, and humerus length), *LRRC27* (fat skatole level), *AVP* (intramuscular fat content, muscle pH), *OXT* (body length, subcutaneous fat thickness), and *FAF1* (intramuscular fat content).

Among the identified candidate genes, *FAF1* has been demonstrated to participate in mouse embryonic development [48]. *LRRC27* DNA methylation shows a significant correlation with weight loss outcomes in humans [49], and *AVP* contributes to muscle pH stability by regulating glucose homeostasis [50]. In the context of adipogenesis, two genes exert distinct effects: *OXT* modulates subcutaneous fat distribution and shows potential relevance for obesity treatment [51, 52], while *PPIA* has been identified as a novel adipogenic factor critically involved in the development of obesity [53]. In neurodevelopment, *ASPM* and *SOX10* are recognized as key regulators. *ASPM* regulates spindle assembly in neural progenitor cells and their proliferation [54, 55], whereas *SOX10* mediates neurodevelopmental processes that influence overall growth dynamics and body conformation in animals [56–58]. Furthermore, *Rab23*, a key regulator of membrane trafficking, supports normal neural tube development by modulating relevant signaling pathways in humans [59].

The GO enrichment analysis of candidate genes revealed a significant enrichment of biological processes related to growth and development, including positive regulation of neuroblast proliferation, developmental growth, cell maturation, and digestive tract morphogenesis, among others. Collectively, these results indicate that the integration of SHAP with the Gsformer model can serve as an interpretable framework to help identify influential SNPs and annotate putative candidate genes.

### Computational cost of Gsformer-NSA versus Gsformer-CSA

Theoretically, Gsformer-NSA introduces sparsity into the attention mechanism, thereby reducing computational overhead compared to Gsformer-CSA. To evaluate this advantage, we compared the training time and peak memory usage of both models on two traits: MY in the cattle dataset and Height in the pig dataset, with the topN hyperparameter set to 20%. Peak memory usage was measured using PyTorch’s torch.cuda.max_memory_allocated() function, which captures the maximum GPU memory allocated by the model during training [60, 61]. All analyses were conducted on a server equipped with eight NVIDIA GeForce RTX 4090 GPUs (8 × 24 GB VRAM). Each analysis was independently repeated 10 times, and the arithmetic mean was reported as the final result to ensure data stability and statistical reliability.

**Fig. 5:**
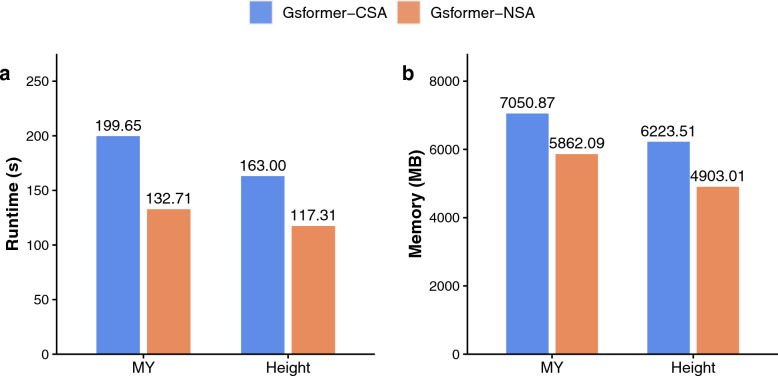
Computational efficiency comparison of Gsformer-CSA vs. Gsformer-NSA for milk yield (MY) and body height (Height). **a** Training time. **b** Peak memory usage during training

As shown in Fig. 5, for the MY trait in the cattle dataset, Gsformer-NSA reduced training time by 33.53% and memory usage by 16.86%. For the Height trait in the pig dataset, training time was reduced by 28.03% and memory usage by 21.22%. These results demonstrate that Gsformer-NSA achieves significantly higher computational efficiency and lower memory demands than Gsformer-CSA.

## Discussion

Genomic selection enhances selection accuracy, shortens generation intervals, and accelerates genetic progress while reducing breeding costs. In recent years, several deep learning models have been developed for GS in plants, demonstrating superior predictive performance over conventional methods. In this study, we introduced Gsformer, a novel deep learning model for phenotypic selection that integrates two distinct feature extraction strategies: CSA, which combines CNNs with a self-attention mechanism, and NSA, which relies on a native sparse attention mechanism. We evaluated the predictive performance of various GS methods using both animal and plant datasets. Gsformer generally ranked first or second in prediction accuracy across all benchmark datasets. Apart from Gsformer, three conventional methods—SVR, GBLUP, and LightGBM—also demonstrated strong performance across scenarios, consistent with previous studies [62–64].

In our study, while marker-level genotypes were employed as genomic features in Gsformer and other methods for most datasets, DNNGP utilizes PCs that capture 95% of the genetic variance explained by the original markers—except in the maize dataset, where PCs were inherently used. This design follows the original DNNGP study [23], in which PCA was adopted for several key reasons: (1) it is a widely used dimensionality reduction (DR) technique; (2) it is well-suited to the high-dimensional input structures that DNNGP was designed to process; and (3) it has been shown to substantially reduce computational time in deep learning models. Notably, the original study reported that DNNGP is fairly robust with or without DR, and in most cases, prediction accuracy tends to improve when using PCs as input. To evaluate the validity of this approach in our comparative analysis, we conducted additional evaluations on the Anx trait from the mouse dataset and the GY3 trait from the wheat dataset. The results showed consistent prediction performance with and without DR—specifically, DNNGP achieved prediction accuracies of 0.181 and 0.200 for Anx, and 0.363 and 0.345 for GY3, before and after PCA-based DR, respectively. These small fluctuations in accuracy suggest that the use of PCs does not meaningfully alter DNNGP’s predictive behavior. Therefore, despite differences in genomic input representations across methods—markers versus transformed PCs—the overall comparison of prediction performance remains valid and fair across methods.

The CSA architecture of Gsformer conducts in-depth feature learning on full feature sequences, enabling robust predictive performance. This design facilitates comprehensive capture and utilization of SNP-level information, maintaining both feature completeness and prediction accuracy. In contrast, Gsformer-NSA introduces sparsity into attention computation, compressing features by reducing redundant interactions. Gsformer-NSA achieved a reduction of more than 20% in both training time and memory usage compared to Gsformer-CSA. With the topN hyperparameter fixed at 20%, Gsformer-NSA performed comparably to or slightly better than Gsformer-CSA for most traits but underperformed for certain traits. Notably, tuning topN fully compensated for Gsformer-NSA’s initial underperformance on these traits—specifically, FP in cattle and DTA in maize—enabling it to match the prediction accuracy of Gsformer-CSA. Given its lower memory consumption, Gsformer-NSA is recommended over Gsformer-CSA when the minor difference in prediction accuracy is acceptable.

Despite its promising results, the CSA architecture has certain limitations. When processing relatively short sequences, the feature extraction capability of CNNs may exceed that of self-attention, as CNNs effectively capture local patterns via convolutional kernels, particularly in sequences with strong local dependencies. Building on this strength, integrating a multi-scale feature learning mechanism—proven effective across deep learning applications [65]—could be beneficial. For instance, PSPNet has significantly improved image classification accuracy by fusing features at four different scales [66], and DGMA2-Net employs a difference-based multi-scale aggregation attention mechanism to enhance change detection in remote sensing imagery [67]. These cases underscore the value of multi-scale feature extraction in boosting model performance in specific contexts. Thus, incorporating such a mechanism into Gsformer-CSA could further enhance its efficacy, improve genomic selection performance, and reduce overall analysis time.

Moreover, the proposed Gsformer model holds considerable potential for applications beyond genomics. It can be adapted to other omics fields, such as transcriptomics and proteomics. Furthermore, a multi-omics fusion learning framework could be developed to integrate data across biological levels. Such an integrative approach would not only improve prediction accuracy but also enhance model interpretability by leveraging complementary information from diverse omics sources.

## Conclusions

In this study, we introduced Gsformer, a novel deep learning framework for phenotypic prediction that incorporates two distinct feature extraction architectures—CSA and NSA. Gsformer generally achieved top-two average prediction accuracy across all six diverse animal and plant datasets. Adjusting the topN value enhanced the performance of Gsformer-NSA, allowing it to match Gsformer-CSA. Due to its lower computational cost, Gsformer-NSA is recommended over Gsformer-CSA in scenarios where a minor compromise in prediction accuracy is acceptable. Furthermore, when combined with SHAP analysis, Gsformer can effectively identify candidate genes associated with traits of interest. In summary, Gsformer establishes a flexible and powerful framework for phenotypic prediction and demonstrates broad applicability in both animal and plant breeding.

## Supplementary Information

Supplementary Material 1. Grid search values for key hyperparameters of machine learning models. Grid search values for key hyperparameters of machine learning models.

Supplementary Material 2. Candidate genes identified to be associated with pig growth and body size traits. Candidate genes identified to be associated with growth and body size traits in pigs.

## Data Availability

The pig dataset used during the current study is available from the corresponding author on reasonable request. Additionally, the datasets for chicken, cattle, mouse, wheat, and maize used in this study were sourced from previously published articles [30–34]. The gene annotation information in GTF format of the pig reference genome *Sus scrofa* 10.2 can be directly downloaded from Ensembl (ftp://ftp.ensembl.org/pub/release-80/gtf/sus_scrofa/). The Gsformer software is available on GitHub at (https://github.com/hajudien/GSformer/tree/master).
